# Adult‐onset dominant muscular dystrophy in Greek families caused by Annexin A11

**DOI:** 10.1002/acn3.51665

**Published:** 2022-09-22

**Authors:** Mridul Johari, George Papadimas, Constantinos Papadopoulos, Sophia Xirou, Aikaterini Kanavaki, Margarita Chrysanthou‐Piterou, Salla Rusanen, Marco Savarese, Peter Hackman, Bjarne Udd

**Affiliations:** ^1^ Folkhälsan Research Center Helsinki Finland; ^2^ Department of Medical and Clinical Genetics, Medicum University of Helsinki Helsinki Finland; ^3^ Department of Neurology, Eginition Hospital, Medical School National and Kapodistrian University of Athens Athens Greece; ^4^ Department of Radiology IASO Children's Hospital Athens Greece; ^5^ Neuromuscular Research Center, Department of Neurology Tampere University and University Hospital Tampere Finland; ^6^ Department of Neurology Vaasa Central Hospital Vaasa Finland

## Abstract

**Objective:**

Mutations in the prion‐like domain of RNA binding proteins cause dysfunctional stress responses and associated aggregate pathology in patients with neurogenic and myopathic phenotypes. Recently, mutations in *ANXA11* have been reported in patients with amyotrophic lateral sclerosis and multisystem proteinopathy. Here we studied families with an autosomal dominant muscle disease caused by *ANXA11*:c.118G > T;p.D40Y.

**Methods:**

We performed deep phenotyping and exome sequencing of patients from four large Greek families, including seven affected individuals with progressive muscle disease but no family history of multi‐organ involvement or ALS.

**Results:**

In our study, all patients presented with an autosomal dominant muscular dystrophy without any Paget disease of bone nor signs of frontotemporal dementia or Parkinson's disease. Histopathological analysis showed rimmed vacuoles with annexin A11 accumulations. Electron microscopy analysis showed myofibrillar abnormalities with disorganization of the sarcomeric structure and Z‐disc dissolution, and subsarcolemmal autophagic material with myeloid formations. Molecular genetic analysis revealed *ANXA11*:c.118G > T;p.D40Y segregating with the phenotype.

**Interpretation:**

Although the pathogenic mechanisms associated with p.D40Y mutation in the prion‐like domain of Annexin A11 need to be further clarified, our study provides robust and clear genetic evidence to support the expansion of the phenotypic spectrum of *ANXA11*.

## Introduction

Causative variants in the low complexity domains (LCDs) of several RNA‐binding proteins have been reported in motor neuron and myogenic disorders.^1^, ^2^, ^3^, ^4^, ^5^, ^6^, ^7^, ^8^ Shared aggregate pathology in multiple post‐mitotic tissues of patients results in these diseases being classified as multisystem proteinopathies (MSPs).^9^, ^10^ In muscle, these pathological accumulations are related to dysfunctional stress granule formation and clearance and result in autophagic rimmed vacuoles.^1^, ^11^, ^12^ Pathogenic mutations in these genes cause both myopathic and neurogenic phenotypes, for example, mutations in the LCD of *HNRNPA1* result in a spectrum of neuromuscular phenotypes caused by shared aggregate pathology.^1^, ^2^ Interestingly, mutations identified and reported to cause ALS have also been observed segregating in families with a primary myopathy with rimmed vacuoles and protein inclusions^3^ or a MSP.^4^

Annexin A11 encoded by *ANXA11* is a member of the calcium‐dependent phospholipid‐binding protein family and is involved in calcium signaling, vesicle trafficking, and apoptosis.^13^, ^14^ Mutations in the *ANXA11* have been reported in familial and sporadic ALS^15^ and recently in MSP.^4^ Previously, a pathogenic c.119A > G:p.D40G variant was identified in the N‐terminal LCD of ANXA11 in European and Korean cohorts of ALS patients.^15^, ^16^ We report four large families from a relatively isolated island in the Greek archipelago with an adult‐late‐onset progressive muscle disease caused by the missense mutation *ANXA11*:c.118G > T:p.D40Y.

## Methods

### Patients and clinical examinations

**Figure 1:**
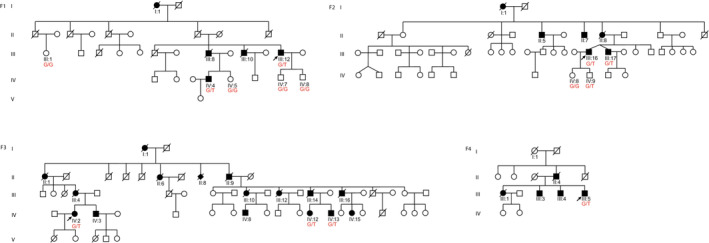
Pedigrees of the four families included in the study. DNA samples were collected from 14 individuals shown with their respective genotype for ANXA11: c.118 G > T in red text.

All patients underwent clinical neuromuscular examination. Besides the seven reportedly affected patients in the four families, we collected blood samples for DNA analysis from seven additional asymptomatic family members (Fig. 1). Patient material for diagnostic purposes was collected after informed consent was obtained from the patients or their legal guardians by the referring clinician (G.P.). The study was performed according to the Declaration of Helsinki, and ethical permission was obtained through the institutional review board (HUS:195/13/03/00/11).

Electrophysiological examination results (nerve conduction studies and needle electromyogram, EMG), creatine kinase measurements, and cardiac function test results were obtained in most patients (Table 1). Echocardiography was performed on five patients.

**Figure 2:**
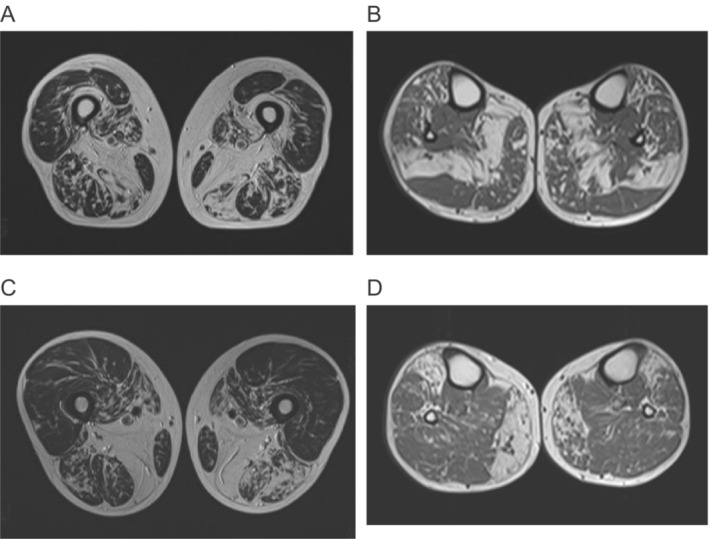
Magnetic resonance imaging (MRI) of F1.III:12 (A‐B) and F1.IV:4 (C‐D). F1.III:2, at age 48 years, showed severe fatty involvement of vastus intermedius and adductor magnus on the thigh with milder fatty involvement in all hamstrings, adductor longus, and vastus medialis (A). The soleus is severely involved with milder changes in tibialis anterior and medial gastrocnemius (B). In the thigh of F1.IV:4 adductor magnus is replaced with milder fatty involvement of hamstrings, vastus medialis, and sartorius (C). On the lower legs, the medial gastrocnemius is asymmetrically, while the tibialis anterior is more symmetrically involved (D).

Muscle MRI findings (Fig. 2) were evaluated in five patients.

**Table 1:** Clinical, histopathological, and MRI details of patients included in the study.

Patient ID	F1.III:12	F1.IV:4	F2.III:16	F3.IV:2	F3.IV:12	F3.IV:13	F4.III:5
Age at onset (years)/first symptoms	52//difficulty in rising arms (R > L)	45//difficulty in raising arms	35//difficulty in raising arms	42//easy fatigability, myalgia	44//difficulty in rising arm	43//difficulty in rising right arm	46/difficulty in rising arms and climbing upstairs
Age at examination/disease duration	60/8 years	50/5 years	51/16 years	58/16 years	55/11 years	53/10 years	56/10 years
Distal upper limb weakness (normal, mild, moderate, or severe) (mild = MRC 4, moderate = 2–3, severe = 0–1)	Mild	No	No	Mild	Mild	No	No
Proximal upper limb weakness (no, mild, moderate, or severe)	Mild	Moderate	Mild	No	Mild	Mild–moderate (asymmetry)	Severe
Proximal lower limb weakness (no, mild, moderate, or severe)	Mild	Moderate	Mild	Mild	No	No	Severe(asymmetry)
Distal lower limb weakness (no, mild, moderate, or severe)	Moderate	Moderate	Moderate	Mild	Mild	Moderate	Severe (asymmetry)
Scapular winging	Prominent	Prominent	Prominent	Prominent	Prominent	Prominent	Yes
Asymmetry of limb weakness yes/no	Yes (mildly asymmetric)	Yes (mildly asymmetric)	Yes (mildly asymmetric)	Yes (mildly asymmetric)	Yes (mild)	Yes	Yes
Walking capacity, when last examined	Unassisted	Unassisted	Unassisted	Unassisted	Unassisted	Unassisted	Wheelchair‐bound
Axial weakness	Mild	Mild	Mild	No	No	No	No
Dropped head	No	No	No	No	No	No	No
Facial weakness	No	No	No	No	Yes	Yes	Yes
Ptosis	Mild	Mild	Mild	Mild	No	No	Prominent
Bulbar symptoms	No	No	No	Mild dysphagia	No	No	No
Respiratory involvement	No	No	No	No	No	No	No
Cardiomyopathy by ultrasound	Mild (EF: 45–50%)	No	Ventricular septal fibrosis (cardiomyopathy) + subendocardial infarction	No	No	No (he has a history of myocardial infarction at the age of 44 yrs)	No
Cataracts	No	No	No	No	No	No	No
Cognitive impairment	No	No	No	No	No	No	No
Paget disease	No	No	No	No	No	No	No
Creatine kinase	Moderately increased (max 713 U/L)	300–1100 U/L	Increased (max 1006 U/L)	Mildly increased (max 410 U/L)	Normal	Mildly increased (max 483 U/L)	Mildly increased (max 541 U/L)
Clinical fasciculations	No	No	No	No	No	No	No
EMG myopathic/neurogenic/mixed	Myopathic	Not performed	Not performed	not performed	not performed	myopathic	mixed
• Spontaneous activity ‐ fibrillations?	No					No	Yes
Histopathology findings General		Not performed	Mild myopathic changes	Myopathic	Not performed	Not performed	Myopathic
• Presence of fiber‐type grouping yes/no			No	No			No
• Rimmed vacuoles yes/no			No	Yes			Yes
Muscle Imaging (MRI) yes/no	Yes	Yes	Yes	Yes	Yes ‐ no pathology	Yes	No
• Most fatty degenerated muscles	Adductors (except left adductor brevis), soleus	Adductors (longus, magnus, semimembranosus, medial gastrocnemius, tibialis ant)	Semimembranosus, semitendinosus, biceps femoris,sartrorius, medial gastrocnemius	Adductor magnus		Adductor magnus and longus (right), short head of biceps, semitendinosus, tibialis ant, medial gastro (L > R)	
• Minor defects	Vastus lateralis, medial gastrocnemius, tibialis ant, long peroneal, long toe extensors	Adductor brevis, vastus lateralis, sartorius, biceps femoris, semitendinosus, soleus, lateral gastrocnemius, long peroneal, long toe extensors	Soleus, lateral gastrocnemius, tibialis ant, long peroneal	Soleus			

### Muscle biopsy, immunohistochemical studies, and imaging

Snap‐frozen muscle samples were obtained from four probands and processed with routine muscle histopathological procedures, including hematoxylin & eosin (H&E), modified Gomori's trichrome, and NADH tetrazolium reductase (NADH‐TR) stainings.^17^ DAB immunostaining was performed using mouse monoclonal anti‐myotilin (clone RSO34, 1:20, LEICA Biosystems Newcastle Ltd, UK) and mouse monoclonal anti‐desmin (clone D33, 1:70, Richard‐Allan Scientific, USA), with Mouse ExtrAvidin Peroxidase Staining Kit (EXTRA2, Merck KGaA, Darmstadt, Germany). Primary antibody anti‐annexin A11 (1:50, polyclonal, Proteintech, cat#10479‐2‐AP, Rosemont, IL, USA) was used with VECTASTAIN® ABC‐HRP Kit, Peroxidase ‐ Rabbit IgG (PK‐4001, Vector Laboratories Inc, USA). Microscopic images were obtained using the NIKON ECLIPSE Ci microscope equipped with an OLYMPUS ColorView II camera. Ultrathin resin sections with a thickness of 70–80 nm were prepared for electron microscopy and examined with an FEI Morgagni 268 transmission electron microscope operating at 80 kV. Electron micrographs were obtained using the Olympus‐SIS Morada digital camera (Olympus Soft Imaging Solutions, Münster, Germany).

### Molecular genetic analyses

Genomic DNA from probands and available family members (Fig. 1) was isolated from blood cells using standard techniques.

F2.III:16, F2.III:17, F3.III:16, F1.III:12, and F3.IV:2 were first screened for known neuromuscular disease‐causing genes via Myocap targeted gene panel sequencing.^18^ Upon initial negative results, F2.III:17 and F1.III:12 underwent Exome Sequencing (ES) using IDT xGen Research Panel v1.0 at Blueprint Genetics (Helsinki, Finland) on Illumina NovaSeq 6000 (100 bp PE); F3.III:16, F3.IV:2, F1.IV:4 and F4.III:5 underwent ES using Agilent SureSelect Human All Exon V6 on DNBSEQ (100 bp PE) while F2.III:16, F2.III:17, F1.III:12, F3.IV:2, F1.IV:8, F1.III:1, F1.IV:4, F1.IV:5, F2.IV:9, F2.IV:8, and F3.IV:13 underwent ES using IDT xGen Exome Research Panel v2 at the BGI Genomics (Hong Kong) on DNBSEQ (100 bp PE). Raw reads were aligned using Burrows‐Wheeler alignment tool (BWA‐MEM) on the UCSC hg38 reference genome, and variants were called according to the Genome analysis tool kit (GATK) recommendations.^19^ Variant annotation was done using Annovar and Ensembl Variant Effect Predictor (VEP). ES results were first filtered on standard quality parameters and then using a minor allele frequency (MAF) ≤ 0.0001 in gnomAD (AF_ex and AF_wgs) database annotated with population frequencies from gnomAD3.0. We used SpliceAI^20^ to predict the potential splicing effects on the mRNA. Validation of *ANXA11* variants was performed by PCR and Sanger sequencing (primers available upon request).

## Results

### Clinical findings

Patients from all four families showed an autosomal dominant skeletal muscle disease. Muscle weakness was first observed in the third to fifth decade in shoulder abduction, particularly in elevating the right arm. Later the symptoms slowly progressed to mild–moderate proximal upper and lower limb weakness and distal lower limb weakness (Table 1). All seven patients presented with prominent scapular winging. Mild ptosis was observed in 4/7 patients while F4.III:5 showed prominent ptosis. Facial weakness or dropped head was not present, but axial trunk weakness was observed in 3/7 patients. No Paget disease of bone nor signs of frontotemporal dementia or Parkinson's disease were recorded, and only one patient showed mild dysphagia. Echocardiography showed mild findings in three individuals (Table 1). Muscle imaging of the patients revealed a characteristic MRI pattern of muscle involvement (Fig. 2) with severe fatty replacement in the adductor magnus and lower leg muscles. Index patients were also tested for FSHD but reported negative.

**Figure 3:**
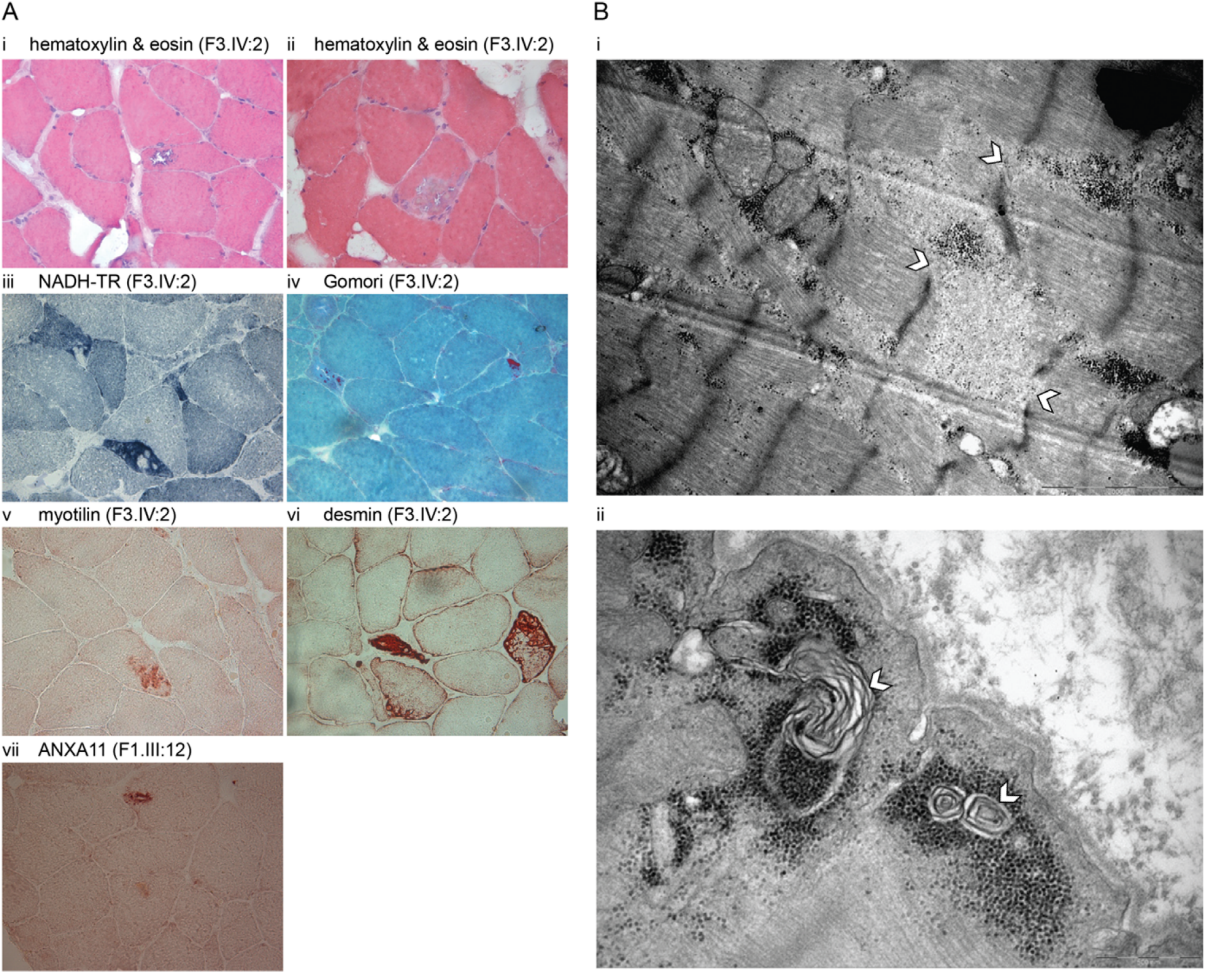
(A) Histochemical and immunohistochemical stainings of left deltoid muscle biopsy sections from F3.IV:2. Hematoxylin & eosin (HE) shows rimmed vacuolated fiber (i) and a larger area of myofibrillar disarray (ii). NADH‐TR staining shows irregular internal architecture with focal areas lacking oxidative activity (iii). Gomori's trichome staining shows red‐purple cytoplasmic inclusions (iv). Abnormal protein accumulations are stained with myotilin (v), larger myofibrillar disarrays with desmin (vi). Staining with anti‐annexin A11 shows positive intravacuolar accumulations (vii). (B) Ultrastructural findings in the muscle biopsy consist of (i) myofibrillar abnormalities with disorganization of the sarcomeric structure and Z‐disc dissolution (shown with white arrowhead), and (ii) subsarcolemmal autophagic material with myeloid formations (shown with white arrowhead).

Besides general myopathic changes, a particular finding in muscle histopathology was the presence of rimmed vacuoles, some myotilin, desmin accumulations (Fig. 3A), and absence of fiber type grouping (Table 1). Staining with Annexin A11 (Fig. 3A vii) revealed accumulations in rimmed vacuolar fibers. Electron microscopy images showed expected myofibrillar alterations and autophagic material (Fig. 3B).

### Molecular genetics

Targeted gene panel analysis via Myocap^18^ was negative for rare variants in already known neuromuscular disease‐causing genes. We analyzed the ES data from all the family members that were exome sequenced (F1‐F4, n = 14). Using the reported affection status and population frequency (MAF ≤0.0001) as criteria for filtering variants, we observed only one variant segregating with the phenotype in all four families. This variant in exon 3 of *ANXA11* (NM_0145869.2) is a single‐nucleotide variant c.118 G > T:p.D40Y, which was not present in any of the reported healthy family members (Fig. 1). One younger presymptomatic family member F2.IV:9 was a carrier. The observed genomic variation was absent in public genome aggregation databases.

## Discussion

We examined seven patients with an adult‐onset autosomal dominant myopathy showing scapuloperoneal pattern of muscle weakness. These patients belonged to five large families on a relatively isolated island of the Aegean Sea. In the following ancestry determination, two families were related and thus merged into one family (F3 in Fig. 1). All patients showed prominent scapular winging and asymmetrical muscle weakness. Mild ptosis was seen in majority of patients while mild axial weakness was observed in some. The combination of asymmetric scapular, distal leg and facial involvement led to a suspicion of FSHD disease but without oral abnormality and orbital weakness. However, the test for FSHD was negative.

Initially, targeted gene panel investigations were negative for all previously known myopathy genes (till 2020). Analysis of ES data from multiple family members identified the c.118G > T:p.D40Y variant in *ANXA11*. This single‐nucleotide variant affects the same amino acid residue 40 as the previously reported pathogenic c. 119A > G:p.D40G found in different cohorts of British and Korean ALS patients.^15^, ^16^ Recently, Leoni and colleagues reported a myopathic MSP in three Brazilian families and the variant c.118G > T:p.D40Y segregating with the phenotype.^4^ However, Teyssou and colleagues also observed the same c.118G > T:p.D40Y variant in two siblings in a cohort of French ALS patients.^21^ These two patients presented with a similar ALS phenotype as observed in individuals carrying the p.G38R variant in that study. But the subsequent biochemical analysis did not provide enough proof for the pathogenicity of p.D40Y compared with p.G38R.^21^ Postmortem tissue analysis of the patient with p.G38R variant showed positive Annexin A11, TDP43, and p62 inclusions in neuropathology studies, but no such evidence was available for the siblings carrying the D40Y variant.^21^

Defects in LCD harboring genes cause various neurological phenotypes and pose a diagnostic challenge for clinicians. We recently reclassified our findings upon examining these two reports and mainly the clinical description of Brazilian families in Leoni et al. As also observed in Leoni et al., this variant D40Y fulfills PM1, PM2, and PM5 criteria according to the ACMG‐AMP guidelines and should be considered “Likely pathogenic.”

Because the variant affects the same amino acid residue previously reported in ALS patients, we carefully examined our patients for any overlapping neurological symptoms (Table 1). Our patients did not have any ALS phenotype as previously in the ALS cohorts^15^, ^16^ or even in the MSP families.^4^ The partly neurogenic EMG findings in one patient with some high‐amplitude motor unit potentials besides the prominent myopathic findings are not enough for coexisting motor neuron disease. The overwhelming myopathic findings mean that the segregating phenotype in these four large Greek families is primarily an autosomal dominant myopathy. Interestingly, our patients had similar age at onset in the third to fifth decade of life, as also reported in Leoni et al. The pattern of muscle involvement on MRI shows myopathic fatty degeneration‐replacement most clearly in the large adductors, semitendinosus, semimembranosus, and gastrocnemius muscles. Histopathological analysis confirmed increased Annexin A11 stainings in rimmed vacuolated fibers of patient biopsies. These findings overlap with those reported in Leoni et al.,^4^ where the authors use the often misreported and problematic term hIBM or hereditary inclusion body myopathy for the MSP phenotype observed in the Brazilian families. It has been suggested that the use of “hIBM” should be avoided prospectively to restrict further confusion with sporadic inclusion body myositis (sIBM).^22^

Our findings show that the mutation ANXA11 c.118G > T:p.D40Y is an apparent pathogenic founder mutation in the Greek archipelago. Since emigration from these islands has been considerable, it can also be expected in other populations with Greek ancestry, although direct relations to the Brazilian families are not currently known. Variants in *ANXA11*, however, should also be prioritized in patients with unsolved primary myopathies.

## Conflicts of Interest

The authors disclose no conflicts of interest.

## Author Contributions

Conceptualization of the study: MJ, MS, PH, and BU. Project administration: MJ, GP, MS, PH, and BU. Funding acquisition: MJ, PH and BU. Supervision: MS, PH, and BU. Patient samples and data collection: GP, CP, SX, and AK. Data analysis and curation: MJ, GP, CP, SX, AK, MC, and SR. Methodology: MJ, GP, CP, SX, SR, MC, and MS. Visualization: MJ, MC, and GP. Writing the original draft: MJ. Review and editing of the manuscript: MJ, GP, MS, PH, and BU.
